# Estimating the prevalence of high-risk drug users using multi-method estimation

**DOI:** 10.3389/fpubh.2026.1878035

**Published:** 2026-08-26

**Authors:** Rožė Perminienė, Natalja Istomina, Rimantas Stukas, Alma Molytė

**Affiliations:** 1 Faculty of Medicine, Institute of Health Sciences, Vilnius University, Vilnius, Lithuania; 2 Faculty of Medicine, Institute of Biomedical Sciences, Vilnius University, Vilnius, Lithuania

**Keywords:** addication, drug, high-risk drug use, injecting drug users, prevalence estimates

## Abstract

**Introduction:**

When conducting studies related to drug use, researchers around the world face the challenge of obtaining the most accurate and reliable data on the extent of drug use, when direct research instruments are difficult to use (direct survey of subjects due to the illegality of the activity is not possible). Problematic high-risk drug use is especially harmful when these substances are used in risky or high-risk ways. Three drug use prevalence studies have been conducted in Lithuania (2006, 2010, 2017); therefore, the latest data on problematic high-risk drug use are extremely relevant. The aim of this study is to assess the prevalence of high-risk drug users using multi-method estimation in Lithuania using 2021 data.

**Results:**

The research hypothesis—the likely increase in the number of high-risk drug users in Lithuania—was confirmed. Over the 5 years (2017–2021), the number of high-risk drug users in Lithuania increased: high-risk opioid users—by 1.6 times, injecting drug users—by 1.7 times. These indicators also increased in Lithuania’s largest cities (Vilnius, Kaunas, Klaipėda)—the most pronounced increase was observed in Vilnius.

**Conclusion:**

The prevalence of high-risk drug use in Lithuania has increased significantly in recent years and is now not only similar to other European countries, but also higher than in some countries. It is also evident that Lithuania has a high number of injecting drug users. The country should be more active in increasing effective preventive and intervention measures for high-risk drug users.

## 1 Introduction

In conducting studies related to drug use, researchers around the world face the challenge of obtaining the most accurate and reliable data on the extent of drug use, when direct research instruments are difficult to use (directly asking subjects about illegality is not possible). The use of narcotic and psychotropic substances as a social and health problem is becoming increasingly complex worldwide (1). Especially harmful is problematic high-risk drug use, which is defined as the use of psychoactive substances (excluding alcohol, tobacco, and caffeine) when these substances are used in a risky (e.g., intensive, continuous) or high-risk way to health (e.g., by injection) (2, 3).

To study problematic high-risk drug use, researchers need to use indirect methods, combining different, often incomplete, data sources to estimate the drug user population indirectly, using statistical extrapolation. Most often, the number of injecting drug users and/or high-risk opioid users in a country in a given year is calculated. Such studies, conducted regularly, ensure continuous and systematic monitoring of the number of injecting drug users in a country and allow for reliable data to be compared with the situation in other countries on this issue. These studies are also important because they provide objective knowledge for evaluating programs, services, and interventions designed to address drug use problems and improve public health. Scientifically based data are useful for shaping the policy of treatment, rehabilitation, and harm reduction services for drug users in a country according to the scale and nature of use. Estimates of the number of high-risk drug users can be used as denominators to assess the capacity, coverage, and quality of services for them, including Needle and Syringe Exchange and Opioid Substitution Treatment programs (3, 4).

In Lithuania, the first study on problem drug use was conducted in 2006, but the estimate of high-risk opioid users was calculated only in Vilnius city. A national-level study was conducted in 2010, using data from 2005 to 2007. The study was conducted using the Capture-Recapture method, which is widely used in conducting such studies. In 2017, a new study on drug use was conducted in Lithuania, conducted by the Republican Centre for Addictive Disorders with foreign partners. The study used data from 2015 to 2016, and various statistical calculation methods were applied; various data sources were used (5–7).

Comparing data from previous drug use studies with data obtained from various sources in 2021, it was hypothesized that the number of high-risk drug users in Lithuania would increase. The aim of this study is to assess the prevalence of high-risk drug users using multi-method estimation in Lithuania using 2021 data.

## 2 Methods

Based on the methodological requirements of the European Monitoring Centre for Drugs and Drug Addiction (EMCDDA) for similar studies conducted both in Europe and Lithuania, researchers distinguished the following research methodology:

The study type is retrospective (observational). The data was obtained from the State Data Agency, in accordance with the Law on the Reuse of Health Data of the Republic of Lithuania (8) (Lietuvos Respublikos pakartotinio sveikatos duomenų naudojimo įstatymas, 2021).

*The target group of the study included*: high-risk drug users, i.e., people aged 15–64, regular users of heroin and other opioids and/or multiple drugs.

The same target population was retained in the study to allow comparison with the 2017 study conducted in Lithuania (“Estimation of the prevalence of high-risk drug users and the coverage of needle and syringe programs and substitution treatment using multi-method calculations in Lithuania in 2015–2016,” conducted by the Republican Centre for Addiction Disorders together with foreign partners). The target age group for this study was 15–64 years. The recommendations of the European Monitoring Centre for Drugs and Drug Addiction were also taken into account (9, 10).

*Sources of research data*: Information system of the Mandatory Health Insurance Fund SVEIDRA; State Register of Deaths and Their Causes.

*Research period (data for certain years)*: from 2021. January 1 to 2021. December 31. In Lithuania, the research was conducted in 2022–2023.

*Research methods*: Indirect methods were chosen for this study based on data availability and quality. The number of high-risk drug users was estimated using the Mortality Multiplier (MM), Capture-Recapture (CRC), and Lincoln–Petersen methods.

The CRC, Lincoln–Petersen approach was used as the primary method to estimate the national and subnational prevalence of high-risk drug users based on linked administrative datasets. The MM method was used as a complementary validation approach to assess the consistency of the prevalence estimates. The final national point estimates were therefore derived from the CRC, Lincoln–Petersen analysis, whereas the MM results were used to evaluate the robustness of the estimates rather than being mathematically combined with them.

The methods used in this study were selected according to the availability and quality of the administrative data. The CRC with the Lincoln–Petersen estimator was used as the primary method because linked administrative datasets were available and allowed estimation of the hidden population based on overlapping records. The MM method was used as a complementary validation approach to assess the consistency of the prevalence estimates. The methodological approach followed the recommendations of the European Monitoring Center for Drugs and Drug Addiction (9–13).

The CRC and Lincoln–Petersen methods were used to estimate the number of high-risk drug users both nationally and in major cities. The latter method provides more reliable estimates of population size than simple comparisons of administrative registers or their averaging. It is more appropriate when it is necessary to estimate the size of a hidden population (addicted individuals, injecting drug users and other hard-to-identify groups) based on overlapping data sources. The essence of the method is based on the analysis of the overlap of two or more independent data sources, which allows for a statistical calculation of the total population size. Meanwhile, the arithmetic mean is an indicator of descriptive statistics, but it does not take into account the overlap of data sources and does not allow for the assessment of individuals who are not included in any of the registers. For this reason, the arithmetic mean is not considered a suitable method for determining the size of hidden or hard-to-reach populations (14–16).

The CRC method was based on the following main assumptions: independence of data sources, closure of the studied population during the observation period, and accurate linking of records between data sources. Data linking between the SVEIDRA information system and the State Register of Deaths and Their Causes was performed using encrypted unique identifiers of individuals provided by the State Data Agency. Since deterministic linking based on a unique identifier was applied (where records were considered to be matched only if the unique identifier was completely identical), the probability of false matches is considered minimal.

In order to comply with the assumption of population closure as much as possible, the analysis was limited to a period of one calendar year (2021). In this way, the influence of migration, the emergence of new users, cessation of use, or other demographic changes on the evaluated indicators was reduced.

The data sources were created and administered for different purposes – for accounting of treatment services and for registering deaths. However, it was not possible to formally assess their independence, therefore this assumption is considered to be only partially met.

The data from the Register of Deaths and Their Causes were collected for the period 2020–2021. The two-year period was chosen in order to calculate a more stable mortality rate for the Mortality Multiplier method and to reduce the influence of random annual fluctuations. The mortality rate was calculated using the combined data for 2020 and 2021, i.e., by dividing the total number of deaths by the total number of observed persons in both years. Applying this method, a mortality rate of 4.01 percent was obtained, which was used in further calculations of the Mortality Multiplier method. In the CRC analysis, only the data for 2021, which corresponded to the SVEIDRA data period, were used.

R version 4.1.2 and Microsoft Excel were used for data analysis.

The study was conducted with the permission of the Vilnius Regional Biomedical Research Ethics Committee on 2023-01-05 a biomedical study No. 2023/1-1,495-954. The State Data Agency received the Permission for Reuse of Health Data No. 5 on 2023-05-05 (Access to the anonymized health data necessary for the study was granted on the State Health Data Reuse Platform from 2023-05-05 to 2023–11-05).

## 3 Results

### 3.1 Data from SVEIDRA

The 2021 SVEIDRA dataset essentially covered the entire national system of treatment provided under state health insurance. In total, 20,781 treatment records related to F11–19 diagnoses were submitted to the SVEIDRA system in 2021 (by gender: 15,313 men (73.7%), 5,468 women (26.3%); by place of permanent residence: 17,710 (85.2%) in the city, 2,924 (14.1%) in the countryside, 147 (0.7%) not specified). The largest number of treatment records were about persons whose place of residence by municipality at the time of treatment was: Vilnius city—9,867, Kaunas city—1,502, Klaipėda city—1,578 persons.

By diagnosis codes, the most common records were F11 (66.1%) (see Table 1).

**Table 1:** Structure of diagnoses according to ICD-10-AM drug use and dependence diagnostic codes.

Diagnosis code	Frequency	Percentages
F11 (opioids)	13,727	66.1
F12 (cannabis)	665	3.2
F13 (sedatives and hypnotics)	3,673	17.7
F14 (cocaine)	58	0.3
F15 (stimulants, e.g., amphetamine)	205	0.9
F16 (hallucinogens)	14	0.1
F18 (volatile substances)	24	0.1
F19 (multiple drugs, may include opioids)	2,415	11.6
Total	20,781	100.0

The SVEIDRA system found that treatment was provided by 267 medical institutions in Lithuania. According to the treatment method, it can be inferred from all records that in 2021 there were 1,176 (5.7%) records for inpatient treatment and 19,605 (94.3%) for outpatient treatment. 8,441 records (40.6%) were found about the specific treatment method used, “substitution treatment with opioid medicinal products.”

This study further excluded diagnoses related to cannabis, sedatives, hallucinogens and volatile solvents (the resulting set would then have 16,347 records). In addition, cocaine-related diagnoses were also excluded due to their small number, as this may represent a different user population (likely not injecting drug users) and may bias the results of the prevalence assessment study. The study was considered comparable to other prevalence assessment studies, where these diagnoses were also excluded from further data processing (e.g., the 2015–2016 prevalence assessment study) (7).

Records of long-term treatment in the SVEIDRA dataset contained repeated episodes per individual (some individuals received treatment multiple times). After data cleaning and duplicate removal from 16,347 records, 16,014 records remained for further analysis: F11 (opioids)—13,407 records (1,236 unique individuals), F15 (stimulants)—204 records (107 unique individuals), F19 (multiple drugs)—2,403 records (919 unique individuals).

The final analysis (based on a unique personal identifier—ID) included 2,262 individuals who were treated for opioid or amphetamine use problems throughout Lithuania.

The analyzed group of individuals was distributed by gender as follows: 1830 men (80.9%), 432 women (19.1%). The average age of the treated individual was 37.9 years (SD ± 9.7, 16–64 years old).

The distribution of persons receiving treatment by permanent residence was as follows: most permanent residents were in the city, 1,809 persons (79.9%), and in the countryside, 415 persons (18.4%).

The largest number of treatment records concerned persons whose place of residence at the time of treatment was: Vilnius city and district municipality together 924 (40.8%), Kaunas city and district municipality together 237 (10.5%), Klaipėda city and district municipality together 233 (10.3%).

According to the treatment method, it can be seen that in 2021, 325 persons (14.4%) received inpatient treatment and 1,937 persons (85.6%) received outpatient treatment. The duration of treatment for 59.7% (1,351 persons) of the persons was 14 days or shorter, and for 40.3% (911 persons)—15 days or more.

### 3.2 Data from the state register of deaths and their causes

The State Register of Deaths and Their Causes contained data on persons whose deaths were related to the use of narcotic or psychotropic substances (i.e., the ICD-10-AM code of the main cause of death and/or substance was included in the list of selected diagnosis codes), selected from death certificates. The data set contained a unique identifier for each person—a specific person ID, which was provided (encrypted) by the State Data Agency.

The registry data provided for 2020/2021 contained 153 records (70 records in 2020, 83 records in 2021) (based on date of death). This study first filtered the data by age, removing individuals who fell outside the study’s age range (15–64 years). In one case, the age was outside this range.

In the next step, the data was filtered by diagnosis. It was found that 2 records had diagnoses that were not suitable for the study and were therefore excluded from further analysis.

Of the 150 records (69 in 2020, 81 in 2021) (by date of death), 19 cases (12.7%) were female, 131 (87.3%) were male. The mean age of the deceased was 41.6 years (SD ± 8.1, 21–64 years).

According to the place of death, it was found that most often these individuals died at home (80 cases, 53.3%), while in some cases (56, 37.4%) the place of death was various (other).

The largest number of deaths was recorded in large municipalities: Vilnius city and district (total) 66 cases (44.0%), Kaunas city and district (total) 21 cases (14.0%), and Klaipėda city and district (total) 14 cases (9.3%).

As can be seen in Table 2, based on the data of the Information system of the Mandatory Health Insurance Fund SVEIDRA and the State Register of Deaths and Their Causes, individual-level data on the prevalence of high-risk drug use in Lithuania were obtained, which will be further used to assess the prevalence of high-risk drug use in Lithuania.

**Table 2:** Individual-level data used to estimate the prevalence of high-risk drug use in Lithuania.

Data source	Local/national	Year	Research method*	Case definition(s) used in the study	N (after data cleaning)	Mean age, years (SD; min-max), % male
State register of Deaths and Their Causes	National	2020/2021	MM, CRC	Registered persons whose deaths were related to the use of narcotic or psychotropic substances	150	41.6 (SD ± 8.1, 21–64), 87.3 percent.
	Vilnius (Vilnius city and district)	2020/2021			66	41.61 (SD ± 7.58, 27–64), 87.9 percent.
Information system of the Mandatory Health Insurance Fund SVEIDRA	National	2021	CRC	Registered persons who received treatment for a drug-related problem (F11, F15 and F19)	16,014 records, 2,262 unique individuals	37.9 (SD ± 9.7, 16–64), 80.9 percent.
				Registered individuals who were treated for an opioid-related problem (F11)	13,407 records, 1,236 unique individuals	41.69 (SD ± 8.20, 19–64), 78.9 percent.

*MM, Mortality Multiplier; CRC, Capture-Recapture Method.

### 3.3 National calculations

The national population estimates of high-risk drug users using the Mortality Multiplier showed that in 2021, 1,236 individuals aged 15–64 were treated for opioid use. Based on the 2021 SVEIDRA dataset, an annual mortality rate of 2.0% was observed (24 deaths among 1,236 treated individuals). This one-year mortality rate is presented for descriptive purposes only and was not used in the final Mortality Multiplier calculations. To obtain a more stable multiplier that is less sensitive to random annual fluctuations, the average mortality rate calculated from the combined 2020–2021 data (4.01%) was used in all subsequent Mortality Multiplier analyses and prevalence estimates.

The proportion of opioid overdose deaths in the total opioid mortality estimated from SVEIDRA data was not known. The EMCDDA pooled analysis of mortality cohort studies among opioid users entering treatment in Europe showed that more than a third (34.9%) of deaths were caused by opioid overdose. Since there are no analogous data for Lithuania, this proportion was extrapolated to Lithuanian conditions. However, it cannot be ruled out that the actual proportion of overdose deaths in Lithuania differs from the European average, and therefore the obtained prevalence estimates may be sensitive to this assumption. This rate was considered the best external parameter available at the time, but its application to the Lithuanian population may be associated with additional uncertainty. It is recommended that sensitivity analyses or the use of national mortality cohort data be performed in future studies. Applying a 4.01% overall mortality rate for Lithuanian opioid users treated under the state health insurance system, this would imply that opioid overdose mortality was around 1.41%.

The 95% binomial confidence interval for the proportion is 1.41% (1.28–2.95%), whereas the normal approximation yields 1.41% (1.2–2.79%). If the n*p > 5 criterion is met, the normal approximation can be used. Using multiplier methods, the target population size was calculated using the formula: given a sample of the population of interest (benchmark) of size B and the probability c that someone from this unknown population will be a member of the sample, the total population T can be estimated using T = B/c [B: the number of identified high-risk drug users (sample or benchmark), in this case the number of deaths from opioid overdose; c: a parameter indicating the probability that a high-risk drug user (unknown target population) will be identified as a member of sample B, in this case the opioid overdose mortality rate (0.0141)].

The SVEIDRA 2021 dataset provided data on 919 individuals treated for polydrug use (F19) and their registered place of residence in Lithuanian municipalities. It is believed that opioids are almost always used in the F19 category in Lithuania.

Vilnius estimates were used to estimate the high-risk drug user population and extrapolated to the national level using information from the SVEIDRA dataset. The inverse Vilnius proportion was used as a multiplier to obtain a preliminary national-level estimate of the high-risk drug user population.

F11 (opioids): 2021 outside Vilnius city 5,501 (41.2%). The inverse proportion of Vilnius was used as a multiplier to obtain a preliminary population of high-risk opioid users at the national level (e.g., 1/0.588 in 2021).
F15 (stimulants): 2021 outside Vilnius city 164 (81.6%). The inverse proportion of Vilnius was used as a multiplier to obtain a preliminary population of high-risk amphetamine users at the national level (e.g., 1/0.184 in 2021).
F19 (multiple drugs): 2021 outside Vilnius city 1750 (73.7%). The inverse proportion of Vilnius was used as a multiplier to obtain a preliminary population of high-risk polydrug users at the national level (e.g., 1/0.263 in 2021).

### 3.4 Subnational calculations

The SVEIDRA dataset contained treatment records, including those of individuals treated for diagnoses F11, F15 and F19. Clients who lived in Vilnius and had undergone a treatment intervention related to one of the aforementioned diagnoses were selected. The State Register of Deaths and their Causes was used as a second data source; matching cases between the two datasets were identified.

A total of 8,159 (59.44%) persons were treated with the diagnosis F11 (opioids) (Vilnius city) in 2021. A total of 12 [0.15, 95% CI (0.08; 0.23), normal approximation] persons died in 2021 from the State Register of Deaths and Their Causes (F11 diagnosis assigned according to the T substance code). A total of 12 [0.15, 95% CI (0.08; 0.26), exact approximation] persons died in 2021 from the death register (F11 diagnosis assigned according to the T substance code).

In total, 38 (18.54%) (Vilnius city municipality) persons were treated with the diagnosis F15 (stimulants) in 2021. In total, there were no deaths with the diagnosis F15 in 2021 (the diagnosis F15 was assigned according to the T substance code).

A total of 629 (26.05%) (Vilnius city municipality) persons were treated with a diagnosis of F19 (multiple drugs) in 2021. A total of 12 [1.91, 95% CI (0.84; 2.98), normal approximation] persons died in 2021 from the death register (F19 diagnosis assigned according to the T substance code). A total of 12 [1.91, 95% CI (0.99; 3.31), exact approximation] persons died in 2021 from the death register (F19 diagnosis assigned according to the T substance code).

The data of overlapping cases from the Kaunas data sets were calculated in a similar way:

In total, 874 (6.37%) persons (Kaunas city municipality) were treated with the diagnosis F11 (opioids) in 2021. In total, there were no deaths with the diagnosis F11 in 2021 (the diagnosis F11 was assigned according to the T substance code).

In total, 33 (16.10%) (Kaunas city municipality) persons were treated with the diagnosis F15 (stimulants) in 2021. In total, 2 [6.06, 95% CI (−2.08; 14.20), normal approximation] persons died in 2021 from the death register (F15 diagnosis assigned according to the T substance code). In total, 2 [6.06, 95% CI (0.74; 20.23), exact approximation] persons died in 2021 from the death register (F15 diagnosis assigned according to the T substance code).

In total, 184 (7.62%) (Kaunas city municipality) persons were treated with a diagnosis of F19 (multiple drugs) in 2021. In total, 5 [2.72, 95% CI (0.37; 5.07), normal approximation] persons died in 2021 from the death register (F19 diagnosis assigned according to the T substance code). In total, 5 [2.72, 95% CI (0.89; 6.23), exact approximation] persons died in 2021 from the death register (F19 diagnosis assigned according to the T substance code).

Data on overlapping cases from the Klaipėda datasets:

In total, 1,237 (9.01%) (Klaipėda city) persons were treated with the diagnosis F11 (opioids) in 2021. In total, 4 [0.32, 95% CI (0.01; 0.64), normal approximation] persons died in 2021 from the death register (F11 diagnosis assigned according to the T substance code). In total, 4 [0.32, 95% CI (0.09; 0.83), exact approximation] persons died in 2021 from the death register (F11 diagnosis assigned according to the T substance code).

In total, 8 (3.90%) (Klaipėda city) persons were treated with the diagnosis F15 (stimulants) in 2021. In total, 1 person from the death register [12.50, 95% CI (−10.42; 35.42), normal approximation] died in 2021 (F15 diagnosis assigned according to the T substance code). In total, 1 person from the death register [12.50, 95% CI (0.32; 52.65), exact approximation] died in 2021 (F15 diagnosis assigned according to the T substance code).

In total, 190 (7.87%) (Klaipėda city) persons were treated with a diagnosis of F19 (multiple drugs) in 2021. In total, 1 person from the death register [0.53, 95% CI (−0.50; 1.56), normal approximation] died in 2021 (F19 diagnosis assigned according to the T substance code). In total, 1 person from the death register [0.53, 95% CI (0.01; 2.90), exact approximation] died in 2021 (F19 diagnosis assigned according to the T substance code).

### 3.5 Aggregate data to assess the prevalence of high-risk drug use

When determining the number and estimates of high-risk drug users, the following target group division was chosen:

- High-risk opioid users (classified as having a diagnosis of F11 (opioids) in the SVEIDRA and death codes T40.0, T40.1, T40.2 and T40.3 in the State Register of Deaths and Their Causes);
- Injection drug users (includes diagnoses of F11 (opioids), F15 (stimulants), F19 (multiple drugs) in the SVEIDRA and death codes T40.0, T40.1, T40.2, T40.3, T43.6, T40.4 and T40.5 in the State Register of Deaths and Their Causes). *Note:* this group also includes persons with a diagnosis of F15 (e.g., amphetamine users) as injecting drug users, because with changing trends in drug use, other drugs are also used by injection, including amphetamines, cocaine, and synthetic cathinones. For example, after analyzing 1,392 used syringes collected in 2020–2021 by the ESCAPE network of 8 European cities, it was found that in 5 cities, half or more of the syringes contained stimulants. Two or more drugs were found in a third of all syringes, indicating that multiple drugs were being used or that injection equipment was being reused, with the most common combination found being a mixture of stimulants and opioids (3).

When comparing the results of this study with previous Lithuanian studies, it is necessary to take into account that different case definitions were applied in different years and different target populations were assessed. Previous studies used the concepts of “problem drug users” or “problem opioid users,” while this study assessed “high-risk opioid users” and “injecting drug users.” Therefore, the data presented in Table 3 should not be interpreted as strictly comparable time series. Direct assessment of trends is the most reliable and was performed by comparing the 2015–2016 and 2021 studies, which used mutually agreed methodologies and target group definitions.

**Table 3:** Comparison of prevalence estimates (95% confidence intervals) reported in previous Lithuanian studies using different methodological approaches and case definitions.*

	2006 study (12)		2010 study (1)			2015/2016 study (19)		2021 study	
	Problem opioid users in 2006	Injecting drug users in 2006	Problem drug users in 2005	Problem drug users in 2006	Problem drug users in 2007	High-risk opioid users in 2015/2016	Injecting drug users in 2015/2016	High-risk opioid users in 2021	Injecting drug users in 2021
Lithuania	4,300	3,200	5,699 (95% CI 5552–5,849)	5,800 (95% CI 5652–5,951)	5,458 (95% CI 5314–5,605)	8,650 (from 4,854 to 12,444)	9,400 (from 8,371 to 10,474)	13,658 (95% CI 13625–13,743)	16,318 (95% CI 16288–16,388)
Vilnius	2,167 (95% CI 1663–2,934)	1,622				1736 (1135–3,447)	3,893 (2755–6,710)	8,054 (95% CI 8023–8,138)	10,735 (95% CI 10698–10,827)
Kaunas						920 (651–1,586)	177 (158–212)	884 (95% CI 879–912)	3,523 (95% CI 3507–3,594)
Klaipėda		750				991 (701–1708)	1778 (1748–1809)	1,237 (95% CI 1235–1,280)	3,855 (95% CI 3847–3,918)

*Different studies used different target populations and case definitions. Therefore, the indicators presented in the table should be considered indicative and are not directly comparable with each other.

The final national point estimates were derived from the Capture–Recapture/Lincoln–Petersen analysis of the linked SVEIDRA and State Register of Deaths and Their Causes datasets. The Mortality Multiplier method was used as an independent validation approach to assess the consistency of the estimates and was not mathematically combined with the final national estimates. Accordingly, the estimated number of high-risk opioid users in Lithuania in 2021 was 13,658 (95% CI 13625–13,743), and the estimated number of injecting drug users was 16,318 (95% CI 16288–16,388). The prevalence rates were: about 7.3 high-risk opioid users and about 8.8 injecting drug users per 1,000 Lithuanian residents aged 15–64 (the actual number of residents aged 15–64 on 1 January 2022 was 1,826,705) (17).

Compared to previous estimates of high-risk drug use in Lithuania (latest available data 2015/2016), over the past 5 years (2017–2021), the number of high-risk drug users in Lithuania has increased: high-risk opioid users by 1.6 times, injecting drug users by 1.7 times. These indicators have also increased in Lithuania’s largest cities (Vilnius, Kaunas, Klaipėda)—the most pronounced increase was observed in Vilnius (see Table 3).

## 4 Discussion

Based on 2021 data, this study estimated that in 2021 there were about 13,658 high-risk opioid users and about 16,318 injecting drug users in Lithuania. Previous estimates of high-risk opioid use in Lithuania showed about 8,650 individuals and 9,400 injecting drug users (based on 2015/2016 data). This shows that the number of high-risk drug users in Lithuania has increased over the recent 5 years and is now not only similar to other European countries, but is already higher than the level of some countries. The current estimated rate of high-risk opioid use in Lithuania (7.3 users per 1,000 Lithuanian residents aged 15–64) is higher than the median estimate for other European countries (3.1 per 1,000 members), based on the latest available EU data. This Lithuanian rate is close to the rates of EU countries such as Austria (6.59 (2021)), Ireland (6.68 (2019)), but lower than the most recent (latest) rates of Finland (7.65 (2017)), Italy (7.42 (2021)), and the United Kingdom (8.42 (2015)). Among the Baltic countries, Latvia was the only country to estimate this rate in 2017, at 5.7 (4.7–7.0) (18).

The rate of injecting drug use in Lithuania (8.8 injecting drug users per 1,000 Lithuanian residents aged 15–64) is also higher than the median estimate for other European countries (2.2 per 1,000 residents), based on the most recent available EU data. This rate for Lithuania is close to that of EU countries such as Finland (7.4 (2017)), the Czech Republic (6.1 (2021)), Latvia (6.1 (2016)), but lower than that of Estonia (10.1 (2015)) (19).

To ensure greater accuracy of the investigation, data were requested from the Departmental Register of Criminal Offences (DRE), which is managed by the Department of Informatics and Communications. However, the required data was not provided to the State Data Agency. Official statistics show that in Lithuania in 2021 under Article 259 of the Criminal Code (Illegal possession of narcotic drugs or psychotropic substances without the purpose of distributing them) 1,517 cases were recorded (in Vilnius—456, in Kaunas—252, in Klaipėda—56) (20). For this reason, the analysis was conducted based on two administrative registers. The inclusion of the Criminal register in the future could improve the coverage of the hidden population and reduce the potential underestimation of the number of high-risk drug users.

It is also worth noting that the data for the study were collected in Lithuania after the new Law on the Reuse of Health Data of the Republic of Lithuania came into force (2022-07-01), when the development of the State Health Data Reuse Platform began. This innovation for health data managers (both providers to the aforementioned platform and managers of that platform) and data recipients from the platform for analysis and processing could have affected the accuracy of the study data. Therefore, once the State Health Data Reuse Platform is fully established and operating effectively and clearly, it is recommended that a similar study be repeated in Lithuania using data from subsequent years.

This study assessed only the prevalence of high-risk drug users. Service coverage, availability, or utilization indicators were not analyzed, so the results of this study cannot be used to directly assess the adequacy of existing treatment or harm reduction services. Additional studies are needed to include coverage indicators for opioid substitution treatment, needle and syringe programs, and other services. The resulting prevalence estimates can be used as denominators in future studies to estimate the coverage of treatment and harm reduction services.

The EMCDDA recommends that prevalence estimates of high-risk drug use be updated regularly and that their methodological comparability across countries be ensured. The frequency of surveys in each country depends on national monitoring systems, data availability and public health needs. Although the EMCDDA has not set a mandatory frequency for surveys of high-risk drug use, a three-to-five-year interval can be considered a methodologically sound compromise between epidemiological sensitivity and survey feasibility. Such a period allows for the accumulation of sufficient register data for capture–recapture analysis, while ensuring that the results obtained remain relevant for public health planning and evaluation of interventions. A three-to-five-year cycle also allows for the monitoring of long-term trends and the comparability of results between different survey periods.

One of the most important assumptions of the Lincoln–Petersen method used in the study is the independence of the data sources. The study used data from two registries, and complete independence may not have been achieved, as the proportion of opioid users in treatment may differ from that of those not in treatment, which may affect the number of registries that overlap. Therefore, the results have been interpreted with caution and presented with confidence intervals.

One limitation of the study is that the confidence intervals reported were calculated based on a statistical model and do not reflect all possible sources of uncertainty. Since the prevalence estimates of high-risk drug users were obtained using indirect methods and administrative registers, the true uncertainty may be larger than the 95% confidence intervals reported. It is recommended that future studies use bootstrap or sensitivity analyses to more fully assess the stability of the estimates.

Another limitation of the study is that there was no possibility to independently assess the quality of data linkage. Although deterministic matching based on a unique identifier is generally considered to be highly accurate and reduces the likelihood of false positive matches, the possibility of isolated false negative matches due to possible inaccuracies or incompleteness of registry data cannot be completely ruled out.

The study used data periods of different lengths: treatment data were analyzed only for 2021, and data for 2020–2021 were used to calculate the mortality rate. This decision was made to reduce the impact of random annual fluctuations on mortality rate estimates. However, it cannot be ruled out that the mortality structure may have differed in different years, which is why this should be considered one of the limitations of the study.

## 5 Conclusion

Our research has shown that the prevalence of high-risk drug use in Lithuania has increased significantly in recent years and is now not only similar to other European countries, but also higher than in some countries. It is also evident that Lithuania has a high number of injecting drug users. The country should be more proactive in increasing the number of effective preventive and intervention measures for high-risk drug users.

## Data Availability

The original contributions presented in the study are included in the article/supplementary material, further inquiries can be directed to the corresponding author.
